# What is the impact of metabolic syndrome and its components on reflux esophagitis? A cross-sectional study

**DOI:** 10.1186/s12876-019-0950-z

**Published:** 2019-02-19

**Authors:** Yi-Hsuan Hsieh, Mei-Fong Wu, Pei-Yu Yang, Wei-Cheng Liao, Yao-Hsuan Hsieh, Yu-Jun Chang, I-Ching Lin

**Affiliations:** 1 Department of Family Medicine, Changhua Christian Hospital, No. 135, St. Nan-Xiao, Changhua City, 500 Taiwan; 2 Department of Health Evaluation, Changhua Christian Hospital, Changhua City, Taiwan; 3 Department of Laboratory, Show Chwan Memorial Hospital, Changhua City, Taiwan; 4 Department of Rehabilitation, Changhua Christian Hospital, Changhua City, Taiwan; 5 Changchun Otolaryngeal Clinic, Chiayi City, Taiwan; 6 Epidemiology and Biostatistics Center, Changhua Christian Hospital, Changhua City, Taiwan; 7 School of Medicine, Chung Shan Medical University, Taichung City, Taiwan; 8 School of Medicine, Kaohsiung Medical University, Kaohsiung City, Taiwan

**Keywords:** Reflux esophagitis, Gastroesophageal reflux, Metabolic syndrome

## Abstract

**Background:**

The prevalence rate of reflux esophagitis (RE) in Asia, including Taiwan, has increased dramatically in recent years. However, few studies have discussed on its relationship with metabolic syndrome (MetS). This study aimed to evaluate the correlation between RE and MetS and its components.

**Methods:**

We conducted a cross-sectional study during 2013 to 2014 in Taiwan. A total of 4895 subjects who completed upper gastrointestinal endoscopy at the Health Examination Center of Changhua Christian Hospital were enrolled. RE was defined according to the upper gastrointestinal endoscopic findings and MetS was defined according to the Taiwanese criteria. Univariate and multivariate logistic regression analyses were applied to calculate odds ratios and 95% confidence intervals for each variable to assess the associated features for RE. We analyzed the relationship between the number of MetS components and the severity of RE using the chi-square test for trend.

**Results:**

The prevalence rates of MetS and RE were respectively 28.5 and 59.6%. According to univariate logistic regression analysis, MetS was significantly associated with RE and remained a positive association in multivariate logistic regression analysis (adjusted OR^ß^ = 1.251; 95% CI = 1.071–1.462; *p* = 0.005). Furthermore, among the five MetS components, elevated blood pressure (adjusted OR^γ^ = 1.163; 95% CI = 1.023–1.323; *p* = 0.021), abdominal obesity (adjusted OR^γ^ = 1.173; 95% CI = 1.020–1.349; *p* = 0.026) and hyperglycemia (adjusted OR^γ^ = 1.306; 95% CI = 1.142–1.495; *p* < 0.001) were positively associated with the presence of RE. A weak association was also found between elevated triglycerides and RE after adjusting for age and gender (adjusted OR^α^ = 1.171; 95% CI = 1.022–1.343; *p =* 0.023). Reduced high-density lipoprotein cholesterol showed no significant difference between groups with and without RE. Older age (≥65 years), male gender, higher body mass index, higher uric acid, smoking, alcohol drinking, and hiatal hernia were found to be significant associated factors for RE. In addition, a dose-response relation between the number of MetS components and the presence of RE was demonstrated in the multivariate analysis. Furthermore, we performed a trend analysis and found the severity of RE got worse as the number of MetS components increased (*p* < 0.001).

**Conclusion:**

This study suggests that MetS is significantly related to the presence and the severity of RE.

## Background

Reflux esophagitis (RE) has become a major public health issue in Asia. A low prevalence rate of 3.3% was reported in an epidemiological report in Singapore by Kang et al. in 1993 [1], however, subsequent studies have reported a rapid increase in Asian populations, ranging from 4.3 to 37.8% [2, 3].

RE is defined as visible esophageal mucosal damage on endoscopy [4, 5]. RE results from the abnormal retrograde flow of gastric contents into the esophagus [5]. RE has been hypothesized to be a multifactorial process, including an abnormal frequency of gastroesophageal reflux, delayed esophageal clearance, delayed gastric emptying, and transient lower esophageal sphincter tone relaxation [6–8]. RE can cause serious complications if untreated for a prolonged period, including esophageal stricture, Barrett’s esophagus, and esophageal adenocarcinoma [4, 5].

Previous studies have reported positive associations between RE and the following associated factors: older age, male gender, smoking, alcohol consumption, and hiatal hernia, and a negative association between RE and *Helicobacter pylori (H. pylori)* infection [9–13]. Obesity is also known to play an important role in RE [9, 10, 14–16]. Further evidences have demonstrated that abdominal obesity, which is the core component of metabolic syndrome (MetS), may be a stronger predictor of RE than obesity [10, 11, 16–20]. MetS is generally recognized to be a combination of metabolic abnormalities, including abdominal obesity, hypertension, hyperglycemia, reduced high-density lipoprotein cholesterol (HDL-C), and elevated triglycerides (TG) [21, 22]. Visceral fat accumulation and insulin resistance are currently thought to be the predominant causes of MetS [6, 21, 23]. Patients with MetS have a high risk of cardiovascular disease, diabetes mellitus, and other atherosclerotic diseases [16, 23, 24]. A retrospective case-control study in China in 2010 found that a high waist-hip ratio, hyperglycemia, hypertriglyceridemia, and MetS were the associated factors for RE, and that HDL-C was associated with a reduced risk of RE in men [25]. Another study in Taiwan in 2017 also found a positive association between MetS and RE [26]. These findings suggest that there may be pathogenetic links between RE and MetS [10, 12, 27].

With the increasing levels of obesity and Western diet in Taiwan, it is important to understand the relationship between MetS and RE and further explore the risk factors for RE. Identification of modifiable risk factors and further prevent RE and MetS earlier in healthy people in Taiwan is an important task. However, there was lack of study related to the correlation between the two diseases in Taiwanese. Therefore, the primary aim of this study was to assess the relationship of MetS and its components and RE. The secondary aim was to investigate the association between the number of MetS components and the severity of RE. We hypothesized that there is a positive association between the two diseases in a Taiwanese population.

## Methods

### Study design and study population

**Fig. 1:**
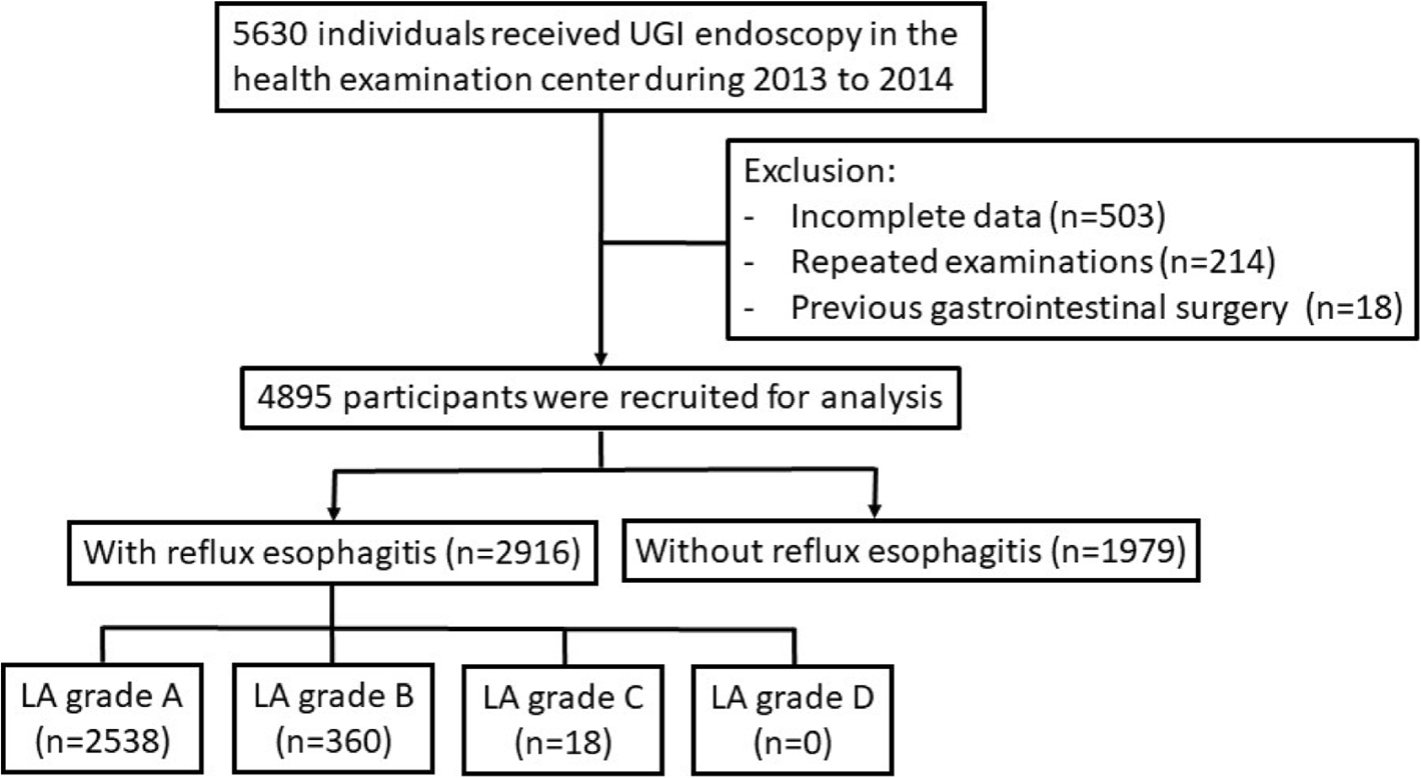
Flow chart of the study design. Abbreviations: *UGI* upper gastrointestinal, *LA grade* Los Angeles classification of esophagitis

In this retrospective cross-sectional study, we enrolled the study population from the Health Examination Center of Changhua Christian Hospital, Changhua City, Taiwan. In total, 5630 subjects who underwent upper gastrointestinal endoscopy at the Health Examination Center from January-2013 to December-2014 were enrolled. Participants with incomplete data (*n* = 503), repeated examinations (*n* = 214), and a clinical history of gastrointestinal surgery (*n* = 18) were excluded. Finally, a total of 4895 participants were recruited for the analysis (Fig. 1). The study protocol was approved by the Ethics Committee of Changhua Christian Hospital (CCH IRB No: 150408).

### Data collection

All 4895 participants underwent upper gastrointestinal endoscopy by experienced gastroenterologists. In addition, all of the enrolled subjects completed a questionnaire of basic data, including age**,** gender, self-reported medical history (including hypertension, diabetes mellitus, hyperlipidemia, and gastrointestinal malignancy), and self-reports of smoking (yes or no), alcohol consumption (yes or no), and betel nut chewing (yes or no). We did not investigate the type or quantity of smoking, alcohol and betel nut consumption in the questionnaire.

All of the participants received physical examinations and measurements of body height, body weight, waist circumference, blood pressure (BP), and body fat percentage. Body mass index (BMI) was calculated as kg/m^2^. The physical examinations were performed by experienced family medicine physicians. Waist circumference (to the nearest 0.1 cm) was measured according to the World Health Organization recommendations: as the midpoint between the lower margin of the least palpable rib and the top of the iliac crest in a standing position [28]. The brachial systolic blood pressure (SBP) and diastolic blood pressure (DBP) were obtained in a sitting position after at least 10 min of resting. We used a body composition analyzer X-Scan plus II (Jawon Medical, Korea) to measure the body fat percentage. In addition, all patients received laboratory blood tests, including fasting plasma glucose (FPG), glycosylated hemoglobin (HbA1C), total cholesterol (TC), TG, low-density lipoprotein cholesterol (LDL-C), and HDL-C. The blood samples were obtained after an overnight fast of at least 8 h.

### Diagnostic criteria

#### Reflux esophagitis

RE was defined according to the upper gastrointestinal endoscopic findings. We divided all of the recruited individuals into two groups: those with RE and those without RE. Furthermore, the subjects with RE were classified into four grades according to the endoscopic finding of the severity of RE. We used the Los Angeles classification of esophagitis (LA) to define the severity of RE as follows [29]:

1. Grade A: one or several mucosal breaks limited to the mucosal folds and each < 5 mm in length.
2. Grade B: one or several mucosal breaks limited to the mucosal folds and at least one mucosal break ≥5 mm in length but not continuous between the tops of two mucosal folds.
3. Grade C: at least one mucosal break extending over two or more mucosal folds, involving < 75% of the circumference.
4. Grade D: One or more mucosal breaks involving ≥75% of the circumference.

#### Hiatal hernia

The diagnosis of hiatal hernia was based on the endoscopic finding, which showed a herniation of abdominal organs through esophageal hiatus of diaphragm into the chest.

#### Helicobacter. Pylori infection

The diagnosis of *H. pylori* infection was based on the rapid urease test. In the process of performing an upper gastrointestinal endoscopy, if the gastroenterologist found a gastric ulcer lesion and felt it was necessary, he/she would perform a mucosa biopsy for rapid urease test.

#### Metabolic syndrome

The Taiwanese criteria for MetS was established based on the 2005 revised National Cholesterol Education Program Adult Treatment Panel III (NCEP ATP III) recommendations for Asians [21]. MetS was defined as the presence of three or more of the following five risk factors:

1. Abdominal obesity: defined as a waist circumference ≥ 90 cm in males and ≥ 80 cm in females.
2. Elevated TG: serum TG ≥150 mg/dL or on drug treatment for elevated TG.
3. Reduced HDL-C: serum HDL-C < 40 mg/dL in males and < 50 mg/dL in females or on drug treatment for reduced HDL-C.
4. Elevated BP: SBP ≥130 mmHg or DBP ≥85 mmHg or receiving treatment for hypertension.
5. Hyperglycemia: FPG ≥100 mg/dL or receiving treatment for diabetes mellitus.

In our study, MetS and its components were defined according to the Taiwanese criteria. Participants with a diabetes history were included in the hyperglycemia group, and those with a hypertension history were included in the elevated BP group. However, we did not include those with a history of hyperlipidemia into elevated TG group or reduced HDL-C group due to lack of information about which lipid-lowering medicine was used.

#### Statistical analysis

Continuous variables were presented as mean ± standard deviation (SD) and categorical variables were presented as number (N) with percentage (%). We analyzed the relationship between the number of MetS components and the severity of RE using the chi-square test for trend. Univariate and multivariate logistic regression analyses were applied to calculate odds ratios (OR) and 95% confidence intervals (CI) for each variable to assess the risk factors for RE. A *p* value < 0.05 was considered to be statistically significant. We used IBM Statistical Package for the Social Sciences (SPSS) version 18.0. for all statistical analyses.

## Results

A total of 4895 participants were recruited for the analysis, with a mean age of 50.1 ± 11.2 years. Of the 4895 participants, 58.4% (*n* = 2859) were male and 41.6% (*n* = 2036) were female. The prevalence rates of MetS and RE were 28.5% (*n* = 1395) and 59.6% (*n* = 2916), respectively. Table 1 presented demographic characteristics and clinical parameters of the study population.

**Table 1:** Demographic characteristics and clinical parameters of the study population (continuous variables)

Characteristics	Reflux esophagitis			Without reflux esophagitis		
	*N*	*Mean*	*SD*	*N*	*Mean*	*SD*
Age (years)	2916	51.20	11.14	1979	48.26	11.04
BMI (kg/m^2^)	2916	24.64	3.54	1979	23.76	3.45
Waist circumference (cm)	2916	82.71	9.95	1979	79.52	9.51
SBP (mmHg)	2916	125.16	16.70	1979	120.96	16.72
DBP (mmHg)	2916	79.77	10.14	1979	77.83	9.90
Body fat percentage (%)	2673	27.19	5.50	1796	27.07	5.58
FPG (mg/dL)	2916	100.52	24.55	1979	96.87	22.24
HbA1C (%)	2747	5.59	0.89	1846	5.45	0.74
TC (mg/dL)	2916	197.59	37.56	1979	196.54	35.79
HDL-C (mg/dL)	2916	48.02	12.30	1979	50.05	12.94
LDL-C (mg/dL)	2914	122.10	32.59	1979	121.29	32.13
TG (mg/dL)	2916	130.70	88.48	1979	115.78	77.96
Uric acid (mg/dL)	2863	6.27	1.48	1938	5.83	1.45

Continuous variables are presented as mean ± *SD; BMI* body mass index, *DBP* diastolic blood pressure, *FPG* fasting plasma glucose, *HbA1C* glycosylated hemoglobin, *HDL-C* high-density lipoprotein cholesterol, *LDL-C* low-density lipoprotein cholesterol, *N* number of patients, *SD* standard deviation, *SBP* systolic blood pressure, *TC* Total cholesterol, *TG* triglycerides

According to univariate logistic regression analysis (Table 2), older age, male gender, smoking, alcohol consumption, betel nut chewing, higher BMI, higher uric acid, hypertension history, diabetes history, *H. pylori* infection, hiatal hernia, elevated TG, abdominal obesity, elevated BP, hyperglycemia, and the presence of MetS were all positively related to RE. On the other hand, hyperlipidemia history, gastric ulcer, and reduced HDL-C showed no significant difference between the groups with and without RE.

**Table 2:** Comparison of associated factors between the participants with and without reflux esophagitis

Variables		Reflux esophagitis	Without reflux esophagitis	*Univariate logistic regression analysis*		
		N (%)	N (%)	*OR*	*95% CI*	*p-value*
Age	< 65 years	2582 (58.4)	1839 (41.6)	1.000		
	≥65 years	334 (70.5)	140 (29.5)	1.699	1.383–2.088	< 0.001**
Gender	Female	1040 (51.1)	996 (48.9)	1.000		
	Male	1876 (65.6)	983 (34.4)	1.828	1.627–2.053	< 0.001**
BMI (mean *± SD*)		24.64 ± 3.54	23.76 ± 3.45	1.076	1.058–1.094	< 0.001**
Uric acid (mean *± SD*)		6.27 ± 1.48	5.83 ± 1.45	1.226	1.178–1.277	< 0.001**
Smoking	No	2402 (57.8)	1753 (42.2)	1.000		
	Yes	514 (69.5)	226 (30.5)	1.660	1.403–1.964	< 0.001**
Alcohol consumption	No	2196 (57.0)	1656 (43.0)	1.000		
	Yes	720 (69.0)	323 (31.0)	1.681	1.453–1.945	< 0.001**
Betel nuts chewing	No	2833 (59.3)	1941 (40.7)	1.000		
	Yes	83 (68.6)	38 (31.4)	1.496	1.015–2.206	0.042*
Hyperlipidemia history	No	304 (63.6)	174 (36.4)	1.000		
	Yes	979 (66.1)	501 (33.9)	1.118	0.902–1.387	0.308
Hypertension history	No	2104 (57.0)	1586 (43.0)	1.000		
	Yes	812 (67.4)	393 (32.6)	1.557	1.358–1.786	< 0.001**
Diabetes history	No	2480 (57.9)	1801 (42.1)	1.000		
	Yes	436 (71.0)	178 (29.0)	1.779	1.479–2.139	< 0.001**
Gastric ulcer	No	2420 (59.4)	1654 (40.6)	1.000		
	Yes	496 (60.4)	325 (39.6)	1.043	0.895–1.216	0.589
*H. pylori* infection^#^	No	2758 (59.2)	1897 (40.8)	1.000		
	Yes	158 (65.8)	82 (34.2)	1.325	1.009–1.741	0.043*
Hiatal hernia	No	2853 (59.1)	1975 (40.9)	1.000		
	Yes	63 (94.0)	4 (6.0)	10.903	3.962–30.002	< 0.001**
Abdominal obesity	No	1983 (57.1)	1490 (42.9)	1.000		
	Yes	933 (65.6)	489 (34.4)	1.434	1.261–1.630	< 0.001**
Elevated TG	No	2065 (57.6)	1521 (42.4)	1.000		
	Yes	851 (65.0)	458 (35.0)	1.369	1.200–1.561	< 0.001**
Reduced HDL-C	No	1856 (58.8)	1302 (41.2)	1.000		
	Yes	1060 (61.0)	677 (39.0)	1.098	0.975–1.238	0.124
Elevated BP	No	1387 (54.6)	1152 (45.4)	1.000		
	Yes	1529 (64.9)	827 (35.1)	1.536	1.369–1.723	< 0.001**
Hyperglycemia	No	1853 (56.1)	1449 (43.9)	1.000		
	Yes	1063 (66.7)	530 (33.3)	1.568	1.384–1.777	< 0.001**
MetS	No	1967 (56.2)	1533 (43.8)	1.000		
	Yes	949 (68.0)	446 (32.0)	1.658	1.455–1.890	< 0.001**
MetS components	0	575 (50.2)	571 (49.8)	1.000		
	1	729 (57.8)	533 (42.2)	1.358	1.156–1.595	< 0.001**
	2	663 (60.7)	429 (39.3)	1.535	1.298–1.815	< 0.001**
	3	533 (68.0)	251 (32.0)	2.109	1.745–2.549	< 0.001**
	4	298 (68.3)	138 (31.7)	2.144	1.699–2.706	< 0.001**
	5	118 (67.4)	57 (32.6)	2.056	1.468–2.879	< 0.001**

OR are calculated by N (%) for categorical variables and by mean ± *SD* for continuous variables using univariate logistic regression analysis. ^#^ Of the 4895 participants who underwent upper gastrointestinal endoscopy, only 719 received rapid urease tests, 240 of which were positive, which represented positive *H. pylori* infection

* *p* < 0.05; ** *p* < 0.001

*BP* blood pressure, *CI* confidence interval, *HDL-C* high-density lipoprotein, *H. pylori* Helicobacter pylori, *MetS* metabolic syndrome, *N* number of patients, *OR* odds ratio, *TG* triglycerides

Table 3 illustrated the comparison of associated features with RE by multivariate logistic regression analysis in three different models. Age and gender were adjusted for each clinical variable in the model 1. The following variables showed positive associations with the presence of RE in the model 1: smoking (adjusted OR^α^ = 1.443; 95% CI = 1.207–1.724; *p* < 0.001), alcohol drinking (adjusted OR^α^ = 1.382; 95% CI = 1.183–1.615; *p* < 0.001), hypertension history (adjusted OR^α^ = 1.201; 95% CI = 1.036–1.393; *p* = 0.015), diabetes history (adjusted OR^α^ = 1.433; 95% CI = 1.182–1.736; *p* < 0.001), hiatal hernia (adjusted OR^α^ = 8.230; 95% CI = 2.978–22.745; *p* < 0.001), abdominal obesity (adjusted OR^α^ = 1.352; 95% CI = 1.186–1.542; *p* < 0.001), elevated TG (adjusted OR^α^ = 1.171; 95% CI = 1.022–1.343; *p* = 0.023), elevated BP (adjusted OR^α^ = 1.197; 95% CI = 1.056–1.356; *p* = 0.005), hyperglycemia (adjusted OR^α^ = 1.306; 95% CI = 1.144–1.492; *p* < 0.001), and the presence of MetS (adjusted OR^α^ = 1.411; 95% CI = 1.233–1.616; *p* = < 0.001).

**Table 3:** Multivariate logistic regression analysis of associated factors and reflux esophagitis

Variables		Model 1			Model 2			Model 3		
		*Adjusted OR* ^*α*^	*95% CI*	*p-value*	*Adjusted OR* ^*ß*^	*95% CI*	*p-value*	*Adjusted OR* ^*γ*^	*95% CI*	*p-value*
Age	< 65 years				1.000			1.000		
	≥65 years				1.649	1.328–2.048	< 0.001**	1.499	1.203–1.868	< 0.001**
Gender	Female				1.000			1.000		
	Male				1.317	1.136–1.527	< 0.001**	1.345	1.159–1.560	< 0.001**
BMI (mean *± SD*)					1.025	1.004–1.047	0.018*			
Uric acid (mean *± SD*)					1.090	1.037–1.145	0.001*	1.096	1.044–1.151	< 0.001**
Smoking	No	1.000			1.000			1.000		
	Yes	1.443	1.207–1.724	< 0.001**	1.272	1.055–1.533	0.012*	1.294	1.072–1.561	0.007*
Alcohol consumption	No	1.000			1.000			1.000		
	Yes	1.382	1.183–1.615	< 0.001**	1.286	1.092–1.514	0.003*	1.263	1.072–1.488	0.005*
Betel nuts chewing	No	1.000								
	Yes	1.211	0.816–1.795	0.342						
Hypertension history	No	1.000								
	Yes	1.201	1.036–1.393	0.015*						
Diabetes history	No	1.000								
	Yes	1.433	1.182–1.736	< 0.001**						
H. pylori infection	No	1.000								
	Yes	1.174	0.890–1.550	0.257						
Hiatal hernia	No	1.000			1.000			1.000		
	Yes	8.230	2.978–22.745	< 0.001**	7.420	2.677–20.567	< 0.001**	7.490	2.699–20.786	< 0.001**
Abdominal obesity	No	1.000						1.000		
	Yes	1.352	1.186–1.542	< 0.001**				1.173	1.020–1.349	0.026*
Elevated TG	No	1.000								
	Yes	1.171	1.022–1.343	0.023*						
Elevated BP	No	1.000						1.000		
	Yes	1.197	1.056–1.356	0.005*				1.163	1.023–1.323	0.021*
Hyperglycemia	No	1.000						1.000		
	Yes	1.306	1.144–1.492	< 0.001**				1.306	1.142–1.495	< 0.001**
MetS	No	1.000			1.000					
	Yes	1.411	1.233–1.616	< 0.001**	1.251	1.071–1.462	0.005*			
MetS components	0	1.000								
	1	1.175	0.996–1.386	0.056						
	2	1.209	1.014–1.441	0.034*						
	3	1.615	1.325–1.968	< 0.001**						
	4	1.603	1.259–2.040	< 0.001**						
	5	1.514	1.072–2.139	0.018*						

Adjusted OR are calculated by N (%) for categorical variables and by mean *± SD* for continuous variables using multivariate logistic regression analysis. ^*α*^ Each variable was adjusted for age and gender in the model 1; ^*ß*^ All variables in the model 2 were controlled by each other; ^*γ*^ All variables in the model 3 were controlled by each other

* *p* < 0.05; ** *p* < 0.001

*BMI* body mass index, *BP* blood pressure, *CI* confidence interval, *H. pylori* Helicobacter pylori, *MetS* metabolic syndrome, *N* number of patients, *SD* standard deviation, *OR* odds ratio, *TG* triglycerides

All variables in the model 2 (including age, gender, BMI, uric acid, smoking, alcohol drinking, hiatal hernia, and MetS) were controlled by each other. The variables that showed strong collinearity with others were excluded from model 2. The results of model 2 showed that MetS remained a significantly positive association with RE (adjusted OR^ß^ = 1.251; 95% CI = 1.071–1.462; *p* = 0.005).

Furthermore, we conducted model 3 to assess the association between RE and the individual components of MetS. All variables in the model 3 (including age, gender, uric acid, smoking, alcohol drinking, hiatal hernia, elevated BP, abdominal obesity and hyperglycemia) were controlled by each other. The variables that showed strong collinearity with others were excluded from model 3. Among the five MetS components, elevated BP (adjusted OR^γ^ = 1.163; 95% CI = 1.023–1.323; *p* = 0.021), abdominal obesity (adjusted OR^γ^ = 1.173; 95% CI = 1.020–1.349; *p* = 0.026) and hyperglycemia (adjusted OR^γ^ = 1.306; 95% CI = 1.142–1.495; *p* < 0.001) remained to be positively associated with the presence of RE.

**Fig. 2:**
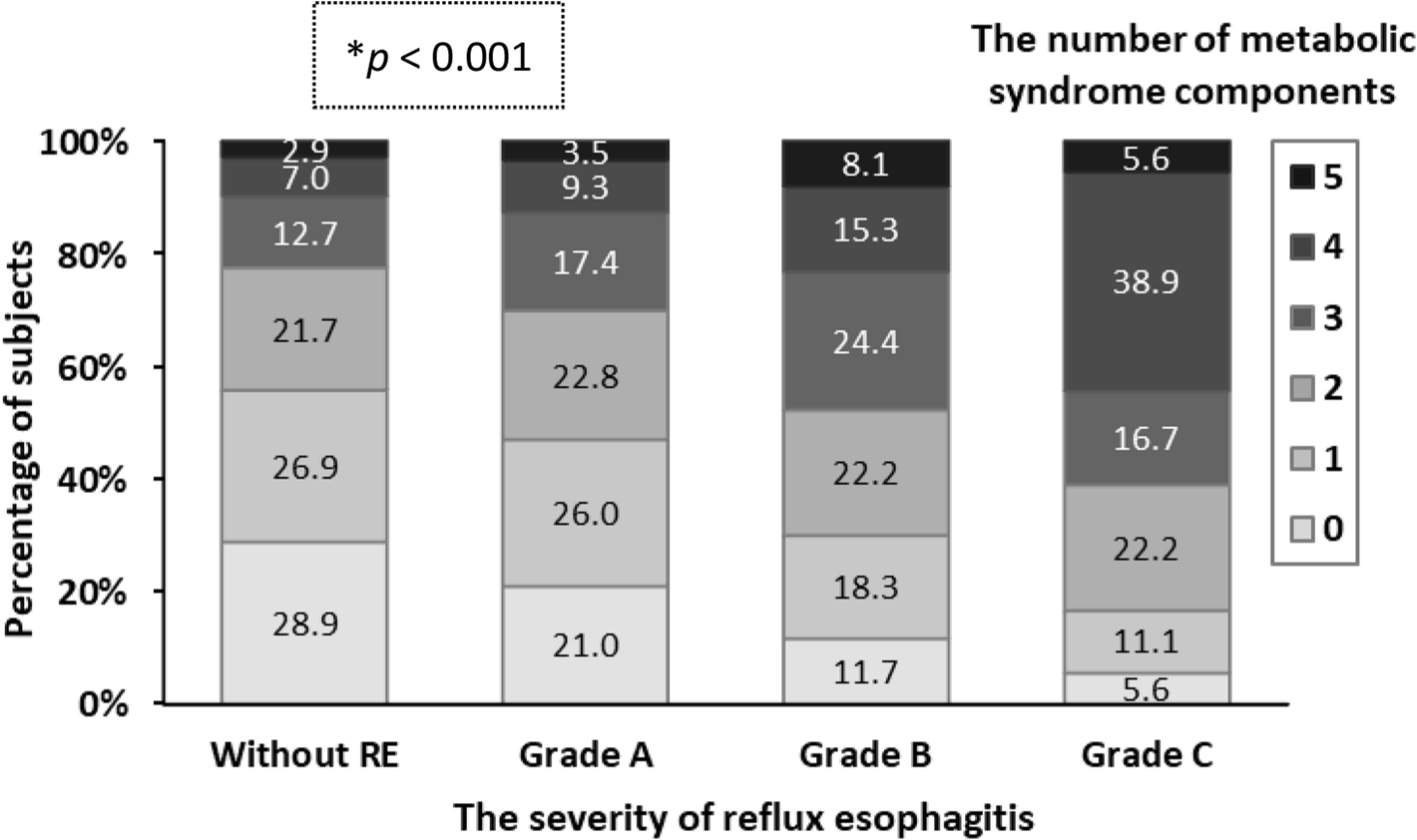
Trend analysis between the severity of reflux esophagitis and the number of metabolic syndrome components. *p* value by the chi-square test for trend. * *p* < 0.001

In the group with RE, the proportion of LA grade A, B, and C were 87.03% (*n* = 2538), 12.35% (*n* = 360), 0.62% (*n* = 18), respectively. There was no participant diagnosed with LA grade D in the present study (Fig. 1). The relationship between the number of MetS components and the severity of RE was analyzed using the chi-square test for trend, as shown in Fig. 2. The trend analysis revealed that the severity of RE was higher as the number of MetS components increased (*p* < 0.001). Table 3 also demonstrated that the adjusted odds ratios of RE increased along with the rising number of MetS components, using 0 component as reference (model 1, Table 3). The results indicated a dose-response relation between the number of MetS components and the presence of RE, which supported the results in the trend analysis.

Take a comprehensive look at the results of model 2 and model 3, older age (≥65 years), male gender, smoking, alcohol consumption, higher BMI, higher uric acid, and hiatal hernia were all found significantly related to RE. Particularly, the presence of hiatal hernia was found to be a strong associated factor for RE.

## Discussion

Same as our initial hypothesis, this cross-sectional population-based study demonstrated that MetS was positively associated with RE. Among the five MetS components, abdominal obesity, elevated BP, hyperglycemia, and elevated TG were independent predictors for RE. Most importantly, this study found a linear trend of association between the number of MetS components and the severity of RE.

Abdominal obesity has been hypothesized to induce gastroesophageal reflux through both mechanical and humoral causes. Abdominal obesity, also called central obesity, is defined as an elevated waist circumference. The significance of an elevated waist circumference is the accumulation of visceral fat. Compared with simple obesity (only defined by BMI), visceral fat accumulation is considered to be a stronger risk factor for the development of hypertension, dyslipidemia, diabetes mellitus, coronary artery disease, and cerebrovascular disease [9]. Visceral fat accumulation has also been reported to increase intra-abdominal pressure and transient lower esophageal sphincter relaxation, which can promote gastroesophageal reflux [9, 15, 19]. In addition to mechanical causes, adipose tissues have been shown to overproduce pro-inflammatory cytokines (interleukin-6, tumor necrosis factor-α, etc.), which can cause esophageal circular muscle relaxation and insulin resistance [9, 19, 24]. These mechanisms can then lead to chronic inflammation of the gastroesophageal junction and result in the development of RE.

Hypertension has been reported to be significantly associated with RE in several previous studies, which is consistent with the our study [9, 30, 31], although the mechanism for this relationship remains unclear. Calcium channel blockers are widely used to treat hypertension. Some studies have reported that calcium channel blockers are important associated factors for RE as they inhibit esophageal muscle contraction and reduce lower esophageal sphincter pressure leading to RE [9, 12, 30]. However, in addition to the side effects of antihypertensive treatment, the influence of hypertension itself must be taken into account. Several studies have suggested that genetic factors may play a role and that environmental factors known to increase blood pressure, such as high salt intake and life stress, may also have an impact on this issue [31]. Since this study is a retrospective cross-sectional study, we did not include environmental factors or exclude the patients using calcium channel blockers in our analysis.

The association between hyperglycemia and RE is controversial. Most previous studies have reported no significant correlation between hyperglycemia and RE [18, 19, 24, 32]. However, an increasing number of studies support a positive relationship between the two diseases [9, 19]. In our study, hyperglycemia (FPG ≥100 mg/dL) was an independent associated factor for RE. Current studies hypothesized that hyperglycemia causes RE due to diabetic autonomic neuropathy and insulin resistance. Vagus nerve injury, a common complication of diabetes autonomic neuropathy, affects esophageal motor and sensory function. Motor dysfunction of the esophagus includes slower gastric emptying, abnormal esophageal motility, ineffective bolus transport, and more frequent esophageal transient lower esophageal sphincter relaxation [9, 30, 33]. The perceptional abnormalities such as nausea and abdominal fullness, which result from esophageal sensory dysfunction, are important in clinical reflux symptoms [33]. Few studies have discussed the occurrence of RE in patients with impaired fasting glucose or glucose intolerance. Some studies have suggested that a high serum glucose level influences the motor function of the esophagus before the diagnosis of diabetes mellitus was made [9, 34]. Our study results strengthen the hypothesis that hyperglycemia is associated with the development of RE.

A weak association was found between elevated TG and RE after adjusting for age and gender (shown in model 1, Table 3). It has been reported that hypertriglyceridemia may be related to RE in several previous studies [9, 19, 26, 30, 35]. Hypertriglyceridemia is known to be associated with high dietary fat. An excessively high fat diet can result in delayed gastric emptying, and further increases the risk of RE [19, 32]. In accordance with our study, most previous studies have not reported a significant association between HDL-C and RE [10, 24, 35].

The most important result of this study is the positive association between MetS and RE. We further identified a linear trend of correlation between the number of MetS components and the severity of RE. According to multivariate analysis, we also found a dose-response relation between the number of MetS components and the presence of RE (shown in model 1, Table 3).

An increase in the number of MetS components was associated with the presence and a more severe grade of RE. This dose-response relation means that the two diseases are not only related to each other, but also have a degree of influence on each other. As described above, most MetS components have been proven associated with the occurrence of RE. A case-control study in Korea in 2008 identified insulin resistance and MetS as risk factors for RE [24]. Moreover, a cross-sectional study in 2011 reported that insulin resistance was an independent predictor for the prevalence and severity of RE [35]. Insulin resistance is currently known as a core factor in the development of MetS. Therefore, it is possible that MetS is an important predictor for the development of RE. From an another point of view, persistent gastric acid stimulation can result in chronic inflammation of the esophagus, and chronic inflammation has been proven to be an important leading cause of MetS [32]. Therefore, it is also possible that RE leads to the occurrence of MetS. Further prospective longitudinal studies are needed to elucidate which MetS or RE occurs first.

We also found different distributions of several baseline characteristics between the groups with and without RE. The individuals with RE were predominant male and exhibited older age, higher BMI, more smoking, and more alcohol consumption. A longitudinal study in 2008 reported possible spontaneous regression in patients with low-grade RE (LA grade A or B) without pharmacological treatment [15]. Accordingly, patients with low grade RE should be encourage to make lifestyle changes to reverse the disease, such as weight reduction, stop smoking and stop drinking alcohol.

There are several limitations to this study. First, previous studies have reported the prevalence of RE ranged from 9.0 to 24.6% in a screening health examination in Taiwan [36]. However, there was a particularly high prevalence rate of RE (59.6%) in our study population. All participants were enrolled from the database of our Health Examination Center, and most of them were able to afford self-paid health examinations, including upper gastrointestinal endoscopy. Although a high socioeconomic status had not been proven to be a causative factor for RE [18], it possibly caused selection bias in our study. Due to the upper gastrointestinal endoscopy was optional, relative invasive and high price, those who already had gastrointestinal symptoms tended to receive it. However, this hypothesis was not confirmed due to lack of asking gastrointestinal symptoms in the initial questionnaire. Overdiagnosis is an another possible explanation of the high prevalence rate of RE in our study population. Most people diagnosed with RE belong to LA grade A (87%). The diagnostic criteria for LA grade A by each gastroenterologist vary widely and may lead to overdiagnosis. Second, this study did not include the effects of several confounding factors, such as lifestyle and dietary habits, co-medications (including aspirin or non-steroid anti-inflammatory drugs, proton-pump inhibitor or histamine-2 receptor antagonists), and socioeconomic status. Excessive fatty sweat food consumption can also weaken lower esophageal sphincter and cause gastroesophageal reflux [8]. Recent aspirin or non-steroid anti-inflammatory drugs use may increase the risk of reflux esophagitis. Otherwise, taking proton-pump inhibitor or histamine-2 receptor antagonists may improve reflux esophagitis [12]. Third, self-reported medical and pharmacology history (such as anti-dyslipidemia medicine use) may result in misclassification bias. Fourth, not everyone received a rapid urease test, so there would be misclassification bias.

There are several strengths to this study. To the best of our knowledge, this is the first study to identify the linear trend of an association between the number of MetS components and the severity of RE. In addition, we enrolled a large number of participants. We also focused on a healthy population so we could suggest early prevention measures for the known risk factors.

## Conclusion

In summary, the present study suggests that MetS is significantly associated with the presence and the severity of RE, although the study could not clarify whether MetS or RE occurred first. Therefore, further prospective longitudinal studies are needed to evaluate the association between RE and MetS in the future.

## Abbreviations

BMI: Body mass index
BP: Blood pressure
DBP: Diastolic blood pressure
FPG: Fasting plasma glucose
*H. pylori*: Helicobacter pylori
HbA1C: glycosylated hemoglobin
HDL-C: High-density lipoprotein cholesterol
LA: Los Angeles classification of esophagitis
LDL-C: Low-density lipoprotein cholesterol
MetS: Metabolic syndrome
RE: Reflux esophagitis
SBP: Systolic blood pressure
TC: Total cholesterol
TG: Triglycerides
